# Diaphragm neurostimulation mitigates the adverse cardiopulmonary effects of positive pressure ventilation

**DOI:** 10.1186/s13054-026-05870-9

**Published:** 2026-02-28

**Authors:** Idunn S. Morris, Thiago Bassi, Andrea Castellvi-Font, Andaleeb Iftikhar, Georgiana Roman-Sarita, Catherine A. Bellissimo, Paweenuch Bootjeamjai, Zhanqi Zhao, Viral Thakkar, Nawzer Mehta, John Granton, Laurent Brochard, Niall D. Ferguson, Ewan C. Goligher

**Affiliations:** 1 Department of Physiology, University of Toronto, ON, Canada; 2 Interdepartmental Division of Critical Care Medicine, University of Toronto, ON, Canada; 3 Critical Care Medicine, Nepean Clinical School, Sydney Medical School, Faculty of Medicine and Health, University of Sydney, Sydney, NSW Australia; 4 Department of Intensive Care Medicine, Nepean Hospital, NSW, Australia; 5 Department of Medicine, Division of Respirology, University Health Network, Toronto, ON, Canada; 6 Lungpacer Medical USA Inc., Exton, PA, USA; 7 Department of Critical Care, Hospital del Mar, Hospital del Mar Research Institute, Barcelona, Spain; 8 Department of Respiratory Therapy, University Health Network, Toronto, ON, Canada; 9 Toronto General Hospital Research Institute, Toronto, ON, Canada; 10 Department of Anesthesiology, King Chulalongkorn Memorial Hospital, Thai Red Cross SocietyFaculty of Medicine, Chulalongkorn University, Bangkok, Thailand; 11 School of Biomedical Engineering, Guangzhou Medical University, Guangzhou, China; 12 Institute of Technical Medicine, Furtwangen University, Villingen‑Schwenningen, Germany; 13 Keenan Centre for Biomedical Research, Li Ka Shing Knowledge Institute, St. Michael’s Hospital, Toronto, ON, Canada; 14 Institute for Health Policy, Management and Evaluation, University of Toronto, Toronto, ON, Canada

**Keywords:** Ventilator induced lung injury, Acute respiratory failure, Diaphragm neurostimulation, Lung protective ventilation, Acute cor pulmonale, Cardiac dysfunction, Hemodynamic performance

## Abstract

**Background:**

Positive pressure ventilation has multiple adverse cardiopulmonary effects leading to impaired lung mechanics and hemodynamic performance. Controlled levels of diaphragm activity may mitigate these. The objectives of the study were to assess the effect of varying levels of diaphragm neurostimulation on respiratory mechanics and hemodynamics during positive pressure ventilation and to establish whether these effects depend on the level of applied positive end-expiratory pressure (PEEP).

**Methods:**

Patients enrolled in a clinical trial of diaphragm neurostimulation underwent a nested randomized cross-over study assessing the effect of passive ventilation alone or in combination with three levels of diaphragm neurostimulation, performed at two levels of PEEP. Respiratory mechanics, distribution of ventilation, and hemodynamics were assessed.

**Results:**

Sixteen patients were included. Diaphragm neurostimulation increased ventilation to the dorsal lung regions in a dose-dependent fashion (median 63% vs. 43% of tidal volume, *p* < 0.001) whereas increasing PEEP had a much more limited effect (median 43% vs. 43%, *p* = 0.05). Diaphragm neurostimulation increased end-expiratory lung volume by 617 mL and decreased respiratory system elastance from 1.4 to 1.2 cm H_2_O/(mL/kg), but only at higher PEEP (*p* = 0.025 and 0.021 for interaction, respectively). Neurostimulation significantly increased cardiac index (2.4 vs. 2.7 L/min/m^2^, *p* = 0.031) without increasing pulmonary artery transmural pressure (*p* = 0.26). In the absence of neurostimulation, increasing PEEP tended to decrease cardiac index (2.4 vs. 2.1 L/min/m^2^, *p* = 0.28).

**Conclusions:**

Diaphragm neurostimulation can mitigate the adverse cardiopulmonary effects of positive pressure ventilation. When combined with higher PEEP, diaphragm neurostimulation enhances dorsal lung recruitment without impairing hemodynamics.

**Trial registration:**

The STIMULUS trial (NCT05465083) was prospectively registered at clinicaltrials.gov (July 2022).

**Supplementary Information:**

The online version contains supplementary material available at 10.1186/s13054-026-05870-9.

## Background

Positive pressure ventilation is the foundation on which management of acute respiratory failure has been based for almost three-quarters of a century. However, positive pressure ventilation has multiple adverse cardiopulmonary effects [1]. First, the rise in intrathoracic pressure depresses venous return, cardiac output, and mean arterial pressure, particularly in the preload-dependent patient [2]. Administering intravenous fluids and vasopressors to counter this effect is associated with additional adverse consequences [3–5]. Second, increasing alveolar pressure increases pulmonary vascular resistance, predisposing to right ventricular dysfunction and cor pulmonale [6, 7]. Third, the combination of sedation and positive pressure ventilation can render the diaphragm inactive, leading to cephalad displacement of the diaphragm particularly in dorsal lung regions [8]. The resulting dorsal lung atelectasis increases intrapulmonary shunt, hypoxic pulmonary vasoconstriction and right ventricular (RV) afterload, and increases lung stress and strain and the risk of ventilator-induced lung injury [9, 10]. Although the use of higher positive end-expiratory pressure (PEEP) might in theory reverse some of these effects, clinical trials to date have not shown benefit, possibly because of variability in the lung recruitment response or further exacerbation of hemodynamic impairment [8]. Finally, diaphragm inactivity due to positive pressure ventilation leads to muscle atrophy and dysfunction, increasing the risk of weaning failure, prolonged ventilation, and associated adverse clinical outcomes [11, 12].

While restoring assisted breathing can reverse many of these adverse effects, excess respiratory drive and effort may cause dyssynchrony, patient self-inflicted lung injury, and diaphragm myotrauma [11, 13–15]. Diaphragm neurostimulation-assisted ventilation is a recently described technique to maintain controlled diaphragm contractions in synchrony with mechanical ventilation [16, 17]. A physiological trial in patients with moderate ARDS demonstrated that diaphragm neurostimulation increases lung compliance and cardiac index [18]. In that study, diaphragm neurostimulation was delivered to achieve moderate diaphragmatic efforts (transdiaphragmatic pressure, 10 cm H_2_O) at a single PEEP level.

Some authors have raised concerns about potential risks of continuous diaphragm neurostimulation during the acute phase of critical illness [19]. Optimal dose selection may be critical to obtain the relevant physiological benefits of diaphragm neurostimulation while minimizing any risks of harm. Moreover, given that the applied PEEP can also affect lung mechanics and hemodynamics, the cardiopulmonary effects of diaphragm neurostimulation may depend substantially on the applied PEEP level.

To characterize the cardiopulmonary effects of varying levels of diaphragm neurostimulation, and to establish whether those effects varied according to the applied PEEP level, we conducted a randomized cross-over trial nested within a previously published clinical trial [20]. We hypothesized that a low or moderate degree of neurostimulation would enhance lung recruitment and improve hemodynamics while avoiding the potential risks of excessive diaphragmatic contractions (i.e., pendelluft, negative alveolar pressure swings). We hypothesized there would be an interaction between PEEP and level of stimulation. Some of these results have been previously reported in the form of a presented but unpublished abstract.

## Methods

### Study design

STIMULUS (NCT05465083) was a single center phase 1 clinical trial evaluating the safety and feasibility of delivering continuous on-demand diaphragm neurostimulation-assisted mechanical ventilation to patients with acute respiratory failure [20]. Twenty passively ventilated participants with acute hypoxemic respiratory failure (AHRF) or post-thoracic surgery (post-pulmonary thromboendarterectomy or bilateral lung transplantation) were enrolled. Transvenous diaphragm neurostimulation was delivered via a specialized electrode–embedded central venous catheter (Lungpacer AeroPace Protect System; Lungpacer Medical Inc, Vancouver, Canada). Stimulations were delivered at a frequency of 15–40 Hz for 0.1–1.2 s in synchrony with each ventilator-delivered breath during controlled ventilation, detected by a pneumotachograph placed in the ventilatory circuit.

As part of the STIMULUS trial, we conducted a nested randomized cross-over trial testing the effect of progressively increasing doses of diaphragm neurostimulation at two levels of positive end-expiratory pressure (PEEP). This study was conducted over a 2-hour period shortly after enrolled patients met criteria to initiate stimulation and device mapping was successfully completed. The protocol for this study was approved by the Research Ethics Board of the University Health Network (22–5815).

### Study procedures

During the titration study, participants were placed in a semi-recumbent position with the transducer for hemodynamic measurement zeroed to atmospheric pressure and placed at the midaxillary level. A belt for electrical impedance tomography (EIT) (PulmoVista 500, Dräger, Lübeck, Germany) and a nasogastric catheter equipped with an esophageal and gastric balloon (NutriVent, Sedana Medical, Mirandola, Italy) were placed. The post-operative group had a pulmonary artery catheter (PAC) in situ for pulmonary artery pressure measurement noting pulmonary capillary wedge pressure was not measured due to the nature of the surgery. Post pulmonary thromboendarterectomy patients had additional continuous cardiac output monitoring (Edwards Lifesciences, Irvine, CA). Participants were ventilated in a volume-cycled mode to maintain consistent tidal volumes across neurostimulation levels, with the exception of participants requiring extracorporeal membrane oxygenation that remained in a pressure-controlled mode while passive.

Patients were randomized to lower or higher PEEP in a cross-over design. Lower PEEP was defined as the lowest level of PEEP tolerated in terms of maintaining peripheral oxygen saturations ≥ 90% at a fraction of inspired oxygen at or close to that set by the clinical team, downtitrated to a minimum of 5–10 cm H_2_O in the surgical and AHRF groups respectively. Higher PEEP was defined as a PEEP sufficient to achieve an end-expiratory transpulmonary pressure ≥ 0 cm H_2_O and at least 5 cm H_2_O higher than the selected lower PEEP level. Diaphragm neurostimulation was sequentially increased in four steps from absent stimulation at baseline (passive ventilation; end-expiratory occlusion pressure [Pocc] = 0 cm H_2_O), followed by stimulation levels targeting Pocc − 5, − 10, and − 15 cm H_2_O. Each step was maintained for 10 min before measurements were obtained. This sequence of steps was repeated at higher and lower levels of PEEP, applied in a random order.

### Study measurements and signal analysis

At each target PEEP and stimulation level, we assessed respiratory mechanics (quasi-static plateau and driving pressures, tidal volume, transdiaphragmatic pressure), hemodynamics (heart rate, mean arterial pressure, vasopressor dosing, and where available, central venous pressure, pulmonary artery pressure, and cardiac index); arterial blood gases (baseline and maximal stimulation levels). EIT recordings were obtained for offline analysis of distribution of ventilation, changes in end-expiratory lung volume, and pendelluft, using previously described methods [21]. Alveolar pressure was computed as the difference between airway pressure and the product of airway resistance and flow. Airway resistance was derived using least-squares multiple linear regression based on the equation of motion, incorporating dynamic airway pressure, flow and volume during inspiration. Minimum alveolar pressure was taken as the lowest value for alveolar pressure during inspiration.

### Analysis

Descriptive statistics were reported using medians (interquartile range, IQR), means (standard deviations, SD), or counts and percentages as appropriate. The effect of stimulation level on the physiological parameters was assessed using linear mixed effects regression. The stimulation level was specified in the model as a single linear term with four levels. Models included a pre-specified term for a potential interaction between neurostimulation level and PEEP. Models of hemodynamic indices were adjusted for norepinephrine dose and vascular pressures measured intrathoracically (pulmonary artery pressure and central venous pressure) were referenced to mean esophageal pressure rather than atmospheric pressure.

A sample size of 15 subjects was computed as sufficient to give 95% power to detect a correlation between diaphragm neurostimulation level and percentage of tidal volume distributed to the dependent lung region with R^2^ ≥ 0.25, based on previous data [22]. This parameter was selected as the endpoint for sample size calculation given that it reflects the predisposition to atelectasis.

## Results

### Study population

Sixteen patients were included in the nested titration study (11 post-surgical patients, 5 patients with AHRF). The other three participants were excluded due to ongoing requirement for neuromuscular blockade, inability to stimulate the diaphragm after initial successful mapping, or inability to recruit a sufficient diaphragm contractile response from stimulation.

Of the 16 included participants, one participant with severe AHRF, dependent on very high levels of PEEP to maintain adequate gas exchange, underwent study procedures at only the high PEEP level. In two participants, the highest target level for diaphragm neurostimulation could not be achieved at maximum neurostimulation. Ventilatory and basic hemodynamic variables were recorded for all 16 participants. Eleven patients had a PAC in situ and eight of these patients had real-time monitoring of cardiac output. Fifteen participants contributed high quality EIT images (not impacted by stimulation artefact). High quality tracings for esophageal pressure were available in 12 patients, and transdiaphragmatic pressure in nine patients.

Participants were predominantly male (81%), with a median age of 60 (IQR 49–64) years (Table 1). Most were admitted to the ICU for post-operative respiratory failure (11, 69%) and had pre-existing lung disease at baseline (14, 88%). A majority had a history of pulmonary hypertension and/or RV dysfunction (9, 57%). One patient was receiving veno-venous extracorporeal membrane oxygenation (ECMO) and two were receiving inhaled nitric oxide for severe AHRF. Excluding the one participant receiving ECMO, PaO_2_/FiO_2_ during passive ventilation (prior to stimulation) was median 305 mm Hg (IQR 147–345) at low PEEP and 286 mm Hg (157–354) at high PEEP. Normalized respiratory system elastance was 1.3 (IQR 1.1–1.6) at low PEEP and 1.4 (1.2–1.8) cm H_2_O/(ml/kg predicted body weight) at high PEEP. The majority of participants were receiving vasoactive support (87% at low PEEP and 80% at high PEEP). Additional clinical characteristics are reported in Table 2 (group-specific characteristics are reported in Table S1).

**Table 1:** Baseline demographics at enrolment (*n* = 16)

Age (years)	60 (49, 64)
Sex; male, n (%)	13 (81%)
Body mass index (kg / m^2^)	27 (25, 35)
Acute respiratory failure type; n (%)	
Acute hypoxemic respiratory failure Post pulmonary thromboendarterectomy Post bilateral lung transplant	5 (31 %) 8 (50 %) 3 (19 %)
Pre-existing respiratory disease; n (%)	
Obstructive lung disease Restrictive lung disease Chronic thromboembolic disease Other Mixed pathology (two or more of above) None or unknown	2 (13 %) 2 (13 %) 5 (31 %) 1 (6 %) 4 (25 %) 2 (12 %)
Cardiac Rhythm; n (%)	
Sinus rhythm Atrial fibrillation	15 (94 %) 1 (6 %)
Pre-existing cardiac dysfunction by echocardiography; n (%)	
Isolated Pulmonary Hypertension	2 (13 %)
Right Ventricular Dysfunction +/- PulmonaryHypertension	7 (44 %)
Biventricular dysfunction	1 (6 %)
Isolated diastolic dysfunction	1 (6 %)
None or unknown	5 (31 %)

Demographics are given as median (interquartile range) or number (percentage) as appropriate

**Table 2:** Baseline physiological variables

	Low PEEP (*n* = 15*)	High PEEP (*n* = 15*)
Total PEEP (cm H_2_O)	8 (5, 10)	14 (11, 16)
End-expiratory Transpulmonary Pressure (cm H_2_O)	0.9 (0.5, 2.6) *n* = 11	3.5 (2.2, 4.7) *n* = 11
Tidal volume (ml/kg PBW)	7 (6, 7)	6 (6, 7)
Respiratory rate (/min)	22 (20, 26)	22 (19, 25)
Peak airway pressure (cm H_2_O)	21 (19, 24)	27 (24, 30)
Plateau airway pressure (cm H_2_O)	16 (15, 19)	23 (20, 25)
Static driving pressure (cm H_2_O)	9 (8, 10)	9 (8, 10)
Normalized respiratory system elastance (cm H_2_O/[ml/kg PBW])	1.3 (1.1, 1.6)	1.4 (1.2, 1.8)
Respiratory system compliance (ml/cm H_2_O)	53 (45, 63)	46 (41, 61)
Extracorporeal Membrane Oxygenation	1 (7%)	1 (6%)
Inspired Nitric Oxide for Hypoxemia	1 (7%)	2 (13%)
FiO_2_*	0.4 (0.4, 0.6) *n* = 14	0.4 (0.4, 0.6) *n* = 14
PaO_2_/FiO_2_ (mm Hg)**	305 (147, 345) *n* = 13	286 (157, 354) *n* = 14
Ventilatory Ratio**	1.6 (1.4, 2.0) *n* = 13	1.7 (1.5, 2.2) *n* = 14
Heart Rate (/min)	82 (73, 89)	84 (70, 88)
Mean Arterial Pressure (mm Hg)	75 (73, 79)	73 (68, 77)
Number of vasoactive infusions; n (%)		
None Single agent Two or more agents	2 (13%) 10 (67%) 3 (20%)	3 (20%) 10 (67%) 2 (13%)
Norepinephrine dose (mcg/kg/min)	0.05 (0.03, 0.09)	0.06 (0.02, 0.10)
Lactate (mmol/L)	1.4 (1.2, 1.8) *n* = 14	1.3 (1.2, 2.0) *n* = 15

Demographics are given as median (interquartile range) or number (percentage) as appropriate. PEEP, positive end-expiratory pressure; PBW, predicted body weight; Pocc, end-expiratory occlusion pressure; FiO_2_, fraction inspired oxygen; PaO_2_, arterial partial pressure oxygen. *Of the 16 included participants, one with severe acute hypoxemic respiratory failure underwent study procedures at the high PEEP level only. Baseline data at low PEEP for another participant was excluded due to the presence of spontaneous respiratory efforts. **One participant receiving veno-venous extracorporeal membrane oxygenation excluded; two participants receiving inspired nitric oxide for acute hypoxemia included

### Diaphragmatic response to neurostimulation

The nested titration study was well-tolerated. Study procedures were not aborted for safety reasons in any patients. The selected higher PEEP level was median 14 cm H_2_O (IQR 11–16) and the selected lower PEEP level was median 8 cm H_2_O (IQR 5–10), with a median difference of 5 cmH_2_O (IQR 5–6; group specific and individual differences in PEEP levels are detailed in Table S1 and Fig. S1 respectively). Pocc became progressively more negative with increasing stimulation (*p* < 0.001, Fig. S2A) with a corresponding dose-related increase in transdiaphragmatic pressure (*p* = 0.001, Fig. S2B). Mean Pocc at each step approximated the target Pocc level.

### Respiratory mechanics and gas exchange

**Fig. 1:**
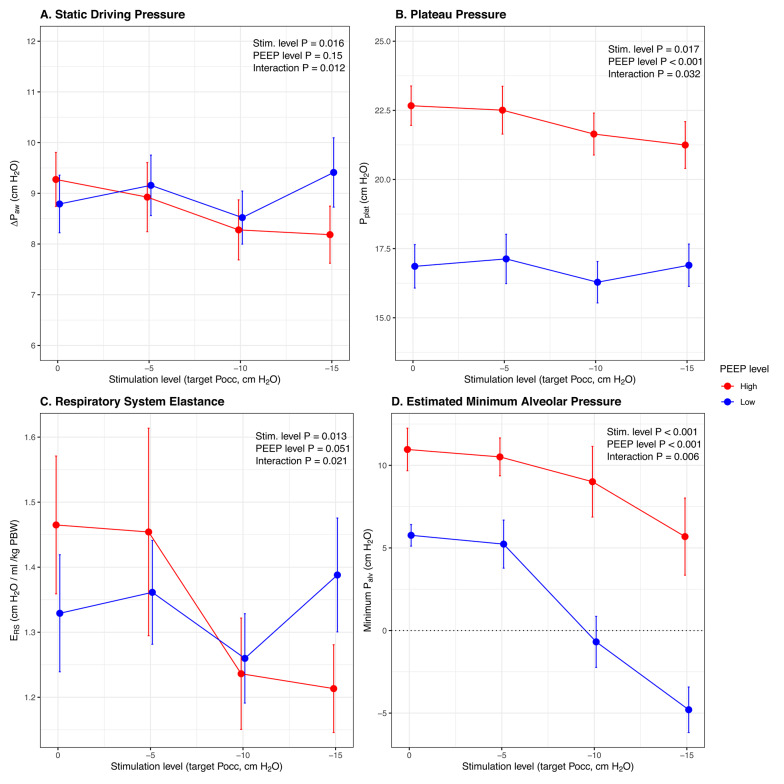
Effect of diaphragm neurostimulation on respiratory mechanics. Mean and standard error bars for static driving pressure (**A**; *n* = 16), plateau pressure (**B**; *n* = 16), normalized respiratory system elastance (**C**; *n* = 16) and estimated minimum alveolar pressure (**D**; *n* = 11), at high (red) and low (blue) PEEP. *ΔP*_*aw*_, *static driving pressure; P*_*plat*_, *plateau pressure; E*_*RS*_, *normalized respiratory system elastance; PBW*,* predicted body weight; P*_*alv*_, *alveolar pressure; Stim.*,* stimulation; PEEP*,* positive end-expiratory pressure; Pocc*,* end-expiratory occlusion pressure*

In the presence of higher PEEP, increasing diaphragm neurostimulation reduced plateau pressure (median 22 cm H_2_O at highest stimulation level vs. 23 cm H_2_O in absence of stimulation, *p* = 0.017), static driving pressure (median 8 cm H_2_O vs. 9 cm H_2_O, *p* = 0.016), and normalized respiratory system elastance (median 1.2 cm H_2_O vs. 1.4 cm H_2_O, *p* = 0.013) (Fig. 1; Table 3). Median respiratory system compliance increased from 46 ml/cm H_2_O to 60 ml/cm H_2_O (*p* = 0.023) (Table 3). These effects were not observed at lower PEEP (p-values for interactions < 0.05, Table 3). The pattern of effects was similar between patient groups (Fig. S3). Individual patient trajectories are shown in Fig. S4.

**Table 3:** Effect of diaphragm neurostimulation on cardiopulmonary parameters

Parameter	PEEP level	Stimulation level: Median (interquartile range)				Statistical significance^a^ (P-values)		
		0 cm H_2_O (none)	–5 cm H_2_O	–10 cm H_2_O	–15 cm H_2_O (maximum)	Effect of higher PEEP^b^	Effect of diaphragm neuro-stimulation^c^	PEEP-neurostimulation Interaction
**ΔP** **(cmH**_**2**_ **O)**	Low	9 (8, 10)	9 (8, 10)	8 (8, 10)	8 (8, 11)	0.15	0.016	0.012
	High	9 (8, 10)	8 (7, 10)	8 (7, 9)	8 (7, 8)			
**P**_**plat**_ **(cmH**_**2**_ **O)**	Low	16 (15, 19)	16 (14, 20)	16 (14, 18)	17 (15, 18)	< 0.001	0.017	0.032
	High	23 (20, 25)	22 (21, 24)	22 (20, 23)	22 (18, 23)			
**E**_**RS, norm**_ **(cm H**_**2**_ **O/ml/kg PBW)**	Low	1.3 (1.1, 1.6)	1.3 (1.2, 1.6)	1.2 (1.1, 1.4)	1.2 (1.2, 1.7)	0.051	0.013	0.021
	High	1.4 (1.2, 1.8)	1.4 (1.0, 1.5)	1.2 (1.0, 1.4)	1.2 (1.1, 1.2)			
**C**_**RS**_ **(ml/cm H**_**2**_ **O)**	Low	53 (45, 63)	50 (46, 61)	57 (47, 66)	54 (43, 63)	0.126	0.023	0.016
	High	46 (41, 61)	50 (44, 67)	57 (50, 71)	60 (49, 71)			
**P**_**alv, min**_ **(cmH**_**2**_ **O)**	Low	5 (5, 7)	5 (3, 8)	−1 (−3, 1)	−5 (−6, −5)	< 0.001	< 0.001	0.006
	High	12 (10, 13)	11 (8, 12)	9 (6, 13)	5 (0, 10)			
**Ventilation to dorsal lung region** **(% V**_**t**_ **)**	Low	43 (40, 49)	53 (46, 59)	61 (56, 67)	62 (52, 65)	0.050	< 0.001	0.97
	High	43 (41, 53)	58 (50, 68)	64 (57, 70)	63 (58, 69)			
**ΔEELV** **(ml)**	Low	Reference	76 (−31, 181)	74 (3, 166)	86 (−131, 154)	< 0.001	< 0.001	0.025
	High	397 (281, 633)	633 (410, 759)	763 (532, 921)	1014 (505, 1137)			
**Pendelluft volume** **(% V**_**t**_ **)**	Low	0 (0, 0)	4 (1, 11)	11 (2, 18)	11 (4, 27)	0.76	< 0.001	0.41
	High	0 (0, 0)	3 (0, 14)	14 (8, 17)	20 (9, 27)			
**Cardiac index** **(L/min/m**^**2**^ **)**^**d**^	Low	2.4 (2.2, 2.6)	2.5 (2.4, 2.8)	2.5 (2.4, 2.6)	2.7 (2.5, 2.9)	0.28	0.031	0.71
	High	2.1 (2.1, 2.5)	2.5 (2.5, 2.7)	2.5 (2.4, 2.7)	2.4 (2.3, 2.9)			
**Heart Rate** **(/min)** ^**d**^	Low	82 (73, 89)	82 (70, 89)	78 (71, 88)	82 (73, 88)	0.87	0.54	0.64
	High	84 (70, 88)	80 (71, 87)	78 (71, 87)	77 (70, 85)			
**Mean arterial pressure** **(mmHg)** ^**d**^	Low	75 (73, 79)	78 (73, 83)	80 (75, 88)	77 (73, 84)	0.021	0.006	0.17
	High	73 (68, 77)	77 (74 78)	77 (75, 80)	76 (75, 78)			
**Mean pulmonary artery transmural pressure** **(mmHg)** ^**d**^	Low	9 (8, 17)	8 (8, 17)	13 (7, 17)	10 (6, 16)	0.71	0.26	0.58
	High	9 (8, 18)	12 (9, 15)	12 (9, 14)	10 (9, 14)			
**Central venous transmural pressure** **(mmHg)** ^**d**^	Low	−3 (−4, 1)	−3 (−4, 1)	−1 (−3, 3)	−2 (−3, 2)	0.83	0.26	0.73
	High	−2 (−3, 3)	0 (−1, 2)	0 (−2, 2)	0 (−1, 3)			

^a^ Computed from linear mixed models incorporating stimulation level, PEEP target, and their interaction as fixed effects, and patient as the random effect

^b^ Referenced to absence of diaphragm stimulation

^c^ Referenced to higher PEEP

^d^ Hemodynamic models are adjusted for noradrenaline dose (cardiac index, heart rate, mean arterial pressure, mean pulmonary artery pressure and central venous pressure). Transmural pressure refers to vascular pressures referenced to mean esophageal pressure to account for differences in pleural pressure

*ΔP*,* static airway driving pressure; Pplat*,* plateau airway pressure; E*_*RS, norm*_, *normalized respiratory system elastance; PBW*,* predicted body weight; C*_*RS*_, *respiratory system compliance; P*_*alv, min*_, *minimum alveolar pressure; V*_*t*_, *tidal volume; ΔEELV*,* change in end-expiratory lung volume (reference; low peep*,* no stimulation)*

Increasing neurostimulation was associated with progressive reductions in minimum alveolar pressure during inspiration (median 5 cm H_2_O vs. 12 cm H_2_O at high PEEP, *p* < 0.001, Fig. 1D; Table 3) and no change in dynamic transpulmonary pressure swing (median 9 cm H_2_O vs. 9 cm H_2_O at high PEEP, *p* = 0.74, Fig. S5). During maximal neurostimulation at low PEEP, the average minimum inspiratory alveolar pressure fell to sub-atmospheric levels (Fig. 1D).

Neither diaphragm neurostimulation nor PEEP level significantly affected PaO_2_/FiO_2_ (*p* = 0.17 and *p* = 0.50 respectively) or ventilatory ratio (*p* = 0.39 and *p* = 0.38 respectively) (Fig. S6).

### Distribution of ventilation

**Fig. 2:**
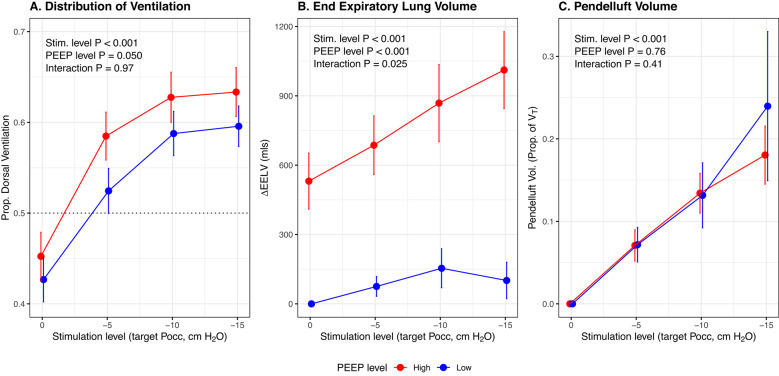
Effect of diaphragm neurostimulation on the distribution of inflation during mechanical ventilation. Mean and standard error bars for proportion of ventilation distributed to dorsal lung regions (**A**; *n* = 15), change in end-expiratory lung volume (**B**; *n* = 15), and pendelluft volume as a proportion of tidal volume (**C**; *n* = 15), at high (red) and low (blue) PEEP. *Prop.*,* proportion;* $\:\varDelta\:$*EELV*,* change in end-expiratory lung volume (reference; low PEEP*,* no stimulation); Vol*,* volume; V*_*T*_, *tidal volume; Stim.*,* stimulation; PEEP*,* positive end-expiratory pressure; Pocc*,* end-expiratory occlusion pressure*

Increasing diaphragm neurostimulation redistributed tidal ventilation to the dorsal lung regions (median 63% vs. 43%, *p* < 0.001, Fig. 2A; Table 3**)**, whereas increasing PEEP had a smaller effect on the distribution of ventilation (median 43% at lower PEEP vs. 43% at higher PEEP, *p* = 0.050). The effect of diaphragm neurostimulation on distribution of ventilation was significantly greater in patients with AHRF compared to post-surgical patients (interaction *p* = 0.012, Fig. S7).

At higher PEEP, increasing diaphragm neurostimulation progressively increased end-expiratory lung volume (median 1014 ml vs. 397 ml, *p* < 0.001, Fig. 2B; Table 3), but not at lower PEEP (median 86 ml vs. reference, interaction *p* = 0.025). Increasing neurostimulation was associated with progressive increases in the volume of ventral-dorsal pendelluft during inspiration (median 20% of tidal volume vs. 0%, *p* < 0.001, Fig. 2C; Table 3); these effects were similar between PEEP levels (interaction *p* = 0.41). Individual patient trajectories are shown in Fig. S8.

### Hemodynamics

**Fig. 3:**
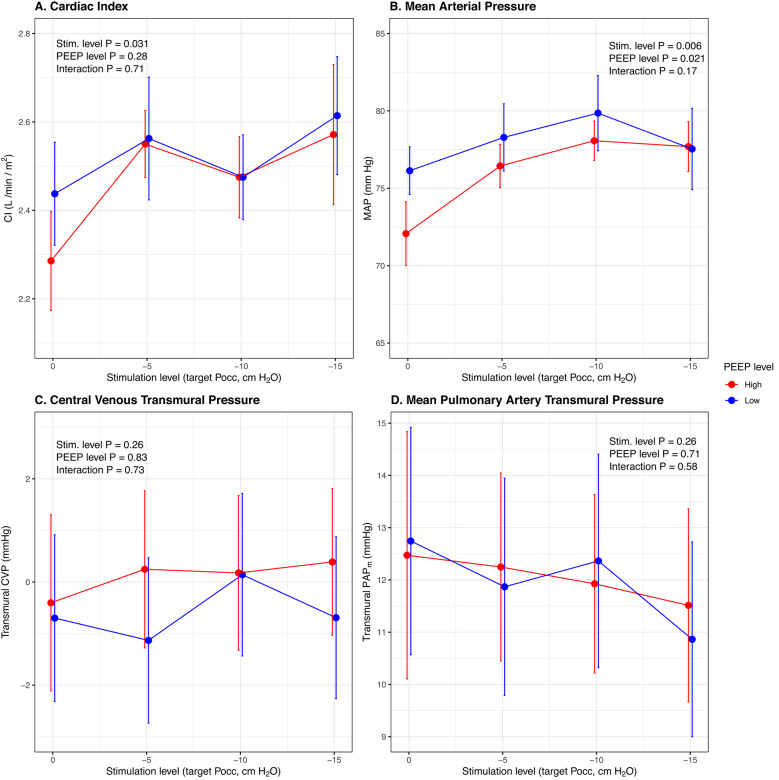
Effect of diaphragm neurostimulation on hemodynamics during mechanical ventilation (statistics adjusted for norepinephrine dose). Central venous transmural pressure and mean pulmonary artery transmural pressure are computed by referencing intravascular pressures to mean esophageal pressure to account for differences in pleural pressure at different neurostimulation levels. Mean and standard error bars for cardiac index (**A**; *n* = 8), mean arterial pressure (**B**; *n* = 16), central venous transmural pressure (**C**; *n* = 9) and mean pulmonary artery transmural pressure (**D**; *n* = 9), at high (red) and low (blue) PEEP. *CI*,* cardiac index; MAP*,* mean arterial pressure; PAP*_*m*_, *mean pulmonary artery pressure; Stim.*,* stimulation; PEEP*,* positive end-expiratory pressure; Pocc*,* end-expiratory occlusion pressure*

Increasing diaphragm neurostimulation increased cardiac index (median 2.4 and 2.7 L/min/m^2^ at highest stimulation level vs. 2.1 and 2.4 L/min/m^2^ in absence of stimulation, at high and low PEEP respectively, adjusted *p* = 0.031, Fig. 3A; Table 3) and mean arterial pressure (median 76 and 77 mm Hg vs. 73 and 75 mm Hg, adjusted *p* = 0.006, Fig. 3B; Table 3) without altering heart rate (adjusted *p* = 0.54, Table 3). Norepinephrine doses were mostly unchanged during study procedures (median 0.06 and 0.04 mcg/kg/min at highest stimulation level vs. 0.06 and 0.05 in absence of stimulation, at high and low PEEP respectively, *p* = 0.63). Central venous transmural pressure and mean pulmonary artery transmural pressure were unchanged (adjusted *p* = 0.26, Fig. 3 and Fig. S9, Table 3). Increasing PEEP reduced mean arterial pressure (median 73 mm Hg at higher PEEP vs. 75 mm Hg at lower PEEP, adjusted *p* = 0.021, Fig. 3B) and this was reversed by diaphragm neurostimulation (Fig. 3B; Table 3). Additional surrogate markers of right ventricular demand and performance are reported in the Supplement (Fig. S10). Individual patient trajectories are shown in Fig. S11.

### Respiratory mechanics during diaphragm neurostimulation versus spontaneous breathing

**Fig. 4:**
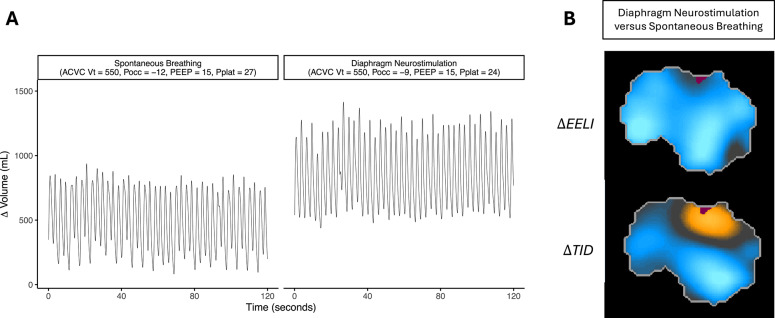
Comparison of spontaneous breathing to passive ventilation with diaphragm neurostimulation in a single participant. **A**. Change in lung volume during spontaneous breathing (left) and diaphragm neurostimulation (right) assessed by electrical impedance tomography. **B**. Electrical impedance tomography images of change in end-expiratory lung impedance (ΔEELI; top) and tidal impedance distribution (ΔTID; bottom) during diaphragm neurostimulation (Pocc − 9 cmH_2_O), referenced to spontaneous breathing (Pocc − 12 cmH_2_O) assessed at PEEP 15 cmH_2_O and tidal volume 550 ml. Blue regions show areas where the parameter is increased. Yellow regions show areas where the parameter is decreased. *EIT*,* electrical impedance tomography; ACVC*,* assist control volume control; Vt*,* tidal volume; Pocc*,* end-expiratory occlusion pressure; PEEP*,* positive end-expiratory pressure; Pplat*,* plateau pressure;* $\:\varDelta\:$*EELI*,* change in end-expiratory lung impedance;* $\:\varDelta\:$*TID*,* change in tidal impedance distribution*

Spontaneous breathing was captured fortuitously at initiation of study procedures in two participants. At constant PEEP, passive ventilation combined with diaphragm neurostimulation was associated with an increase in end-expiratory lung volume as compared to spontaneous breathing with similar (Fig. 4) or greater (Fig. S12) levels of inspiratory effort.

## Discussion

In this study, increasing diaphragm neurostimulation was associated with a progressive increase in diaphragm contractility. These diaphragmatic contractions reversed the adverse cardiopulmonary effects of positive pressure ventilation: ventilation was re-distributed to the dorsal lung regions and hemodynamic performance improved. Some effects of diaphragm neurostimulation were dependent on the applied PEEP: at higher PEEP, diaphragm neurostimulation increased lung volume and reduced driving pressure and respiratory system elastance, consistent with some degree of lung recruitment. By the same token, higher PEEP had a much more favorable effect on respiratory mechanics in the presence of diaphragm neurostimulation. At the same time, vigorous diaphragm contractions from neurostimulation elicited potentially adverse cardiopulmonary effects including increased regional lung stress (as reflected by ventral-dorsal pendelluft) and large negative swings in alveolar pressure (which may predispose to hydrostatic edema formation [23]). Isolated diaphragm contraction alters thoracoabdominal motion, and alongside changes in diaphragm geometry can alter the relationship between transdiaphragmatic pressure and Pocc. We conclude that diaphragm neurostimulation can reverse or attenuate many of the adverse cardiopulmonary effects of positive pressure ventilation and enhance the physiological benefit and tolerability of applying higher PEEP, but optimal dose selection and concomitant PEEP management may be critical to maximizing clinical benefit from this technique.

In a seminal paper, Froese and Bryan demonstrated that relaxation of the diaphragm during mechanical ventilation and general anesthesia results in dorsal atelectasis [8]. Replicating the work of Parfait et al., we found that diaphragm neurostimulation reverses this effect and redistributes ventilation dorsally [18]. The effect of neurostimulation on lung mechanics depended on the applied PEEP level. In the presence of higher PEEP, diaphragm neurostimulation progressively increased end-expiratory lung volume while decreasing respiratory system elastance and driving pressure, consistent with a lung recruitment response. Conversely, at lower PEEP, diaphragm neurostimulation had a greatly attenuated effect on end-expiratory lung volume and did not significantly affect respiratory mechanics, suggesting that little recruitment was obtained. Increases in dorsal ventilation were more pronounced in the patients with AHRF, likely because they had greater atelectasis at baseline.

The same physiological interaction suggests that diaphragm neurostimulation may facilitate a beneficial effect of PEEP on lung mechanics. The goal of PEEP is to recruit the lung and reduce atelectasis, but the degree of lung recruitment with increased PEEP varies widely between patients, and this variability may account for the lack of definitive benefit in clinical trials of higher PEEP ventilation strategies. We found that diaphragm neurostimulation substantially augmented the effect of PEEP on the distribution of ventilation and on end-expiratory lung volume, possibly by alleviating the weight of the abdomen on the lung (analogous to the previously described effects of prone positioning or continuous negative abdominal pressure) [24]. At the same time, diaphragm neurostimulation mitigated the adverse effect of higher PEEP on hemody­namic performance. By the same token, higher PEEP appeared to potentiate the effect of diaphragm neurostimulation, possibly by stabilizing lung recruitment and preventing derecruitment during the expiratory phase. Higher PEEP may also stiffen the chest wall and prevent inward motion of the rib cage, thereby enhancing the effect of diaphragm contraction on lung volume. However, even at high PEEP, no change in dynamic transpulmonary pressure swing was observed, possibly owing to regional increases in lung volume and flow-resistive pressure masking reductions in elastic pressure.

These observations suggest that diaphragm neurostimulation combined with higher PEEP might serve to safely and effectively recruit the injured or atelectatic lung, thereby potentially reducing the risk of ventilator-induced lung injury. Consistent with this hypothesis, driving pressure and respiratory system elastance were reduced by the combined effects of higher PEEP and neurostimulation. However, it is important to note that the effect sizes are small –possibly owing to the mild severity of disease at baseline– and thus require further evaluation in future clinical trials that include patients with more severely impaired respiratory mechanics. Of note, oxygenation and ventilatory ratio were unchanged in response to both neurostimulation and PEEP in this study, possibly due to mild impairment in gas exchange at baseline or the short time interval from intervention to sampling.

Several mechanisms could account for the observed increase in cardiac index with diaphragm neurostimulation. Positive pressure ventilation reduces the gradient for venous return by increasing pleural pressure and mitigating the cyclic increase in intra-abdominal pressure resulting from diaphragmatic contractions. By eliciting diaphragm contractions, neurostimulation may reverse this effect. The increase in cardiac index and mean arterial pressure reached a relative plateau at moderate-to-high doses of neurostimulation, possibly mirroring the ceiling-effect observed in the Frank-Starling relationship. Since pulmonary artery transmural pressures were unchanged despite increasing cardiac output, neurostimulation could have reduced pulmonary vascular resistance and RV afterload, possibly by lowering alveolar pressure and improving pulmonary inflation. These effects could have important clinical benefits for patients in shock or those at risk of acute cor pulmonale. However, these observations were obtained in a small number of patients in this study, all of whom were post-operative, and further investigation is required to definitively characterize the hemodynamic effects of diaphragm neurostimulation, particularly among patients with AHRF.

These data provide important insights on dose selection for a diaphragm neurostimulation-assisted ventilation strategy. Concerns have been raised about the possibility of load-induced diaphragm injury in the presence of sarcolemmal hyperfragility and diaphragm myofibrillar hibernation [25]. Our present observations suggest that higher levels of stimulation could also injure the lung by inducing regional lung stress (manifesting as pendelluft) and negative alveolar pressures; avoiding these adverse effects by maintaining a low-to-moderate stimulation level may optimize the balance of benefit and harm. One approach might be to apply intermittent high dose stimulations (‘sigh breaths’) to facilitate lung recruitment while mostly maintaining low dose stimulation. Stimulation could also be titrated to improve hemodynamic performance in shock. The effect of prolonged low dose stimulation on diaphragm function is uncertain; in our previous paper from the STIMULUS trial where patients were subjected to prolonged stimulation, diaphragm function was generally preserved, which may be reassuring [20]. Randomized trials are needed to establish the balance of benefit and harm with this technique.

We incidentally observed in two patients that spontaneous breathing was associated with a lower lung volume compared to diaphragm neurostimulation at equivalent PEEP levels, suggesting an important difference in respiratory mechanics between spontaneous breathing (expiratory muscles active) and diaphragm neurostimulation-assisted ventilation (expiratory muscles quiescent). Expiratory muscle effort during spontaneous breathing can decrease lung volume and increase atelectasis and hypoxemia. Abolishing expiratory muscle effort with neuromuscular blockade can increase end-expiratory lung volume in patients with ARDS [26]. While spontaneous breathing has many salient benefits in reversing the adverse effects of positive pressure ventilation and may accelerate recovery in relatively less severely ill patients (e.g. PaO_2_/FiO_2_ >150 mm Hg) [27], high respiratory drive and high levels of expiratory muscle effort may exacerbate lung injury and hypoxemia when lung mechanics and gas exchange are seriously impaired (e.g., PaO_2_/FiO_2_ <150 mm Hg). In this case, controlled ventilation combined with diaphragm neurostimulation may be safer and better tolerated approach to achieving lung- and diaphragm-protective targets.

A number of limitations should be noted. This study consisted of a small sample size, of primarily surgical patients, who exhibited less severe respiratory failure than medical counterparts. The generalizability of the findings is therefore somewhat limited, although they represent fundamental physiological phenomena. Furthermore, certain physiological variables were only available from a homogenous cohort (e.g. cardiac output in patients post pulmonary thromboendarterectomy) which may limit the generalizability of our findings to other groups. The consistency of our results with previous studies is reassuring in terms of the effect of diaphragm neurostimulation on both respiratory and hemodynamic profiles. While our observations suggest a potential reduction in RV afterload, lack of pulmonary capillary wedge pressure measurement and/or echocardiographic assessment limits the conclusiveness of this finding. Finally, as a physiological study, manipulations in PEEP may not have been clinically indicated and the clinical benefit of a higher PEEP strategy coupled with diaphragm neurostimulation cannot be determined from this study.

## Conclusions

Diaphragm neurostimulation can reverse many adverse cardiopulmonary effects of positive pressure ventilation: it redistributes tidal ventilation to the dorsal lung regions and improves hemodynamic performance. Diaphragm neurostimulation combined with higher PEEP increased lung volume and improved respiratory mechanics, suggesting a synergistic effect on lung recruitment. Using diaphragm neurostimulation to target low-to-moderate levels of diaphragmatic effort in combination with a higher PEEP strategy may optimally balance the benefits of improving ventilatory homogeneity, lung recruitment, and cardiovascular function, while avoiding the potential harms of vigorous diaphragm contractions on regional lung stress and alveolar pressures.

## Supplementary Information

Supplementary Material 1

## Data Availability

Deidentified data are available upon reasonable request one year after publication.

## Abbreviations

AHRF: Acute Hypoxemic Respiratory Failure
ARDS: Acute Respiratory Distress Syndrome
ECMO: Extracorporeal Membrane Oxygenation
EIT: Electrical Impedance Tomography
FiO2: Fraction of Inspired Oxygen
ICU: Intensive Care Unit
IQR: Interquartile Range
PAC: Pulmonary Artery Catheter
PaO2: Arterial Partial Pressure of Oxygen
PEEP: Positive End-Expiratory Pressure
Pocc: End-Expiratory Occlusion Pressure
RV: Right Ventricle
SD: Standard Deviation

## Declarations

Prospective written consent was obtained from eligible participants undergoing elective surgery, and prospective substitute decision-maker consent was obtained on behalf of eligible participants receiving invasive mechanical ventilation to manage acute hypoxemic respiratory failure.
