# Supplementary material for: Diaphragm neurostimulation mitigates the adverse cardiopulmonary effects of positive pressure ventilation
Source: Crit Care. 2026 Feb 28;30:182. doi: 10.1186/s13054-026-05870-9 (PMC13085342; doi:10.1186/s13054-026-05870-9)
Supplement: Supplementary file 1 — Supplementary Material 1 [file 13054_2026_5870_MOESM1_ESM.pdf]

ONLINE SUPPLEMENT

Diaphragm Neurostimulation Mitigates the Adverse Cardiopulmonary Effects of Positive Pressure Ventilation

Idunn S Morris, Thiago Bassi, Andrea Castellvi-Font, Andaleeb Iftikhar, Georgiana Roman-Sarita, Catherine A Bellissimo, Paweenuch Bootjeamjai, Zhanqi Zhao, Viral Thakkar, Nawzer Mehta, John Granton, Laurent Brochard, Niall D Ferguson, and Ewan C Goligher

CONTENTS

Table S1. Baseline physiological variables by group. 2

Fig. S1. Positive End-Expiratory Pressure by patient group. 3

Fig. S2. Relationship between the level of diaphragm neurostimulation and magnitude of inspiratory effort. 4

Fig. S3. Effect of diaphragm neurostimulation on respiratory mechanics by patient group. 5

Fig. S4. Effect of diaphragm neurostimulation on respiratory mechanics at high and low PEEP for individual patients. 7

Fig. S5. Effect of diaphragm neurostimulation on measured dynamic transpulmonary driving pressure. 9

Fig. S6. Effect of diaphragm neurostimulation on gas exchange. 10

Fig. S7. Effect of diaphragm neurostimulation on distribution of inflation during mechanical ventilation by patient group. 11

Fig. S8. Effect of diaphragm neurostimulation on distribution of inflation at high and low PEEP for individual patients. 13

Fig. S9. Comparison of intravascular versus transmural central venous pressure and mean pulmonary artery pressure. 14

Fig. S10. Effect of diaphragm neurostimulation on surrogate measures of right ventricular demand and performance. 15

Fig. S11. Effect of diaphragm neurostimulation on hemodynamics at high and low PEEP for individual patients. 17

Fig. S12. Comparison of spontaneous breathing to passive ventilation without diaphragm neurostimulation, and passive ventilation with diaphragm neurostimulation in a single participant. 19

Table S1. Baseline physiological variables by group.

	AHRF Group		Surgical Group	
	**Low PEEP (n=4)**	**High PEEP (n=5)**	**Low PEEP (n=11)**	**High PEEP (n=10)**
Total PEEP (cm H_2O)	10 (9, 10)	16 (15, 16)	6 (5, 10)	12 (10, 15)
End-expiratory Transpulmonary Pressure (cm H_2O)	1.0 (1.0, 1.1)<br>*n=2*	3.5 (3.0, 3.7)<br>*n=3*	0.7 (0.5, 2.7)<br>*n=9*	3.1 (1.6, 5.8)<br>*n=8*
Tidal volume (ml /kg PBW)	6 (6, 6)	6 (5, 6)	7 (6, 7)	7 (6, 7)
Respiratory rate (/min)	21 (18, 23)	22 (20, 24)	22 (20, 28)	23 (19, 26)
Peak airway pressure (cm H_2O)	23 (21, 25)	30 (29, 34)	21 (18. 24)	27 (24, 29)
Plateau airway pressure (cm H_2O)	18 (17, 18)	23 (23, 24)	15 (15, 18)	21 (20, 24)
Static driving pressure (cm H_2O)	9 (8, 9)	9 (7, 9)	9 (8, 11)	9 (8, 10)
Normalized respiratory system elastance (cm H_2O/[ml /kg PBW])	1.3 (1.1, 1.5)	1.6 (1.2, 1.8)	1.3 (1.1, 1.6)	1.3 (1.1, 1.7)
Respiratory system compliance (ml /cm H_2O)	54 (50, 62)	42 (40, 71)	50 (45, 63)	48 (43, 58)
Extracorporeal Membrane Oxygenation	1 (25 %)	1 (20 %)	0 (0 %)	0 (0 %)
Inspired Nitric Oxide for Hypoxemia	0 (0 %)	1 (20 %)	0 (0 %)	0 (0 %)
FiO_2*	0.60 (0.60, 0.65)<br>*n=3*	0.65 (0.60, 0.73)<br>*n=4*	0.40 (0.40, 0.43)	0.40 (0.40, 0.44)
PaO_2/FiO_2 (mm Hg)*	133 (126, 140)<br>*n=2*	134 (121, 157)<br>*n=4*	313 (233, 364)	305 (281, 369)
Ventilatory Ratio*	1.9 (1.8, 2.1)<br>*n=3*	2.0 (1.6, 2.4)<br>*n=4*	1.5 (1.3, 1.9)	1.7 (1.4, 1.9)
Heart Rate (/min)	72 (58, 82)	73 (63, 84)	88 (74, 90)	86 (74, 88)
Mean Arterial Pressure (mm Hg)	75 (73, 76)	74 (69, 77)	76 (73, 82)	73 (68, 76)
Number of vasoactive infusions; n (%)				
None	1 (25 %)	3 (60 %)	1 (9%)	0 (0 %)
Single agent	3 (75%)	2 (40 %)	7 (64 %)	8 (80 %)
Two or more agents	0 (0 %)	0 (0 %)	3 (27 %)	2 (20 %)
Norepinephrine dose (mcg/kg/min)	0.05 (0.03, 0.10)	0.00 (0.00, 0.06)	0.05 (0.03, 0.09)	0.07 (0.03, 0.11)
Lactate (mmol/L)	1.3 (1.0, 1.8)<br>*n=3*	1.3 (0.6, 1.9)<br>*n=5*	1.5 (1.3, 1.8)<br>*n=10*	1.5 (1.2, 2.1)<br>*n=10*

Baseline demographics are measured at no stimulation. Demographics are given as median (interquartile range) or number (percentage) as appropriate.

** Excludes participant on veno-venous extracorporeal membrane oxygenation (AHRF) but not participants receiving inspired nitric oxide for acute hypoxemia.*

PEEP, positive end-expiratory pressure; PBW, predicted body weight; FiO_2, fraction of inspired oxygen; PaO_2, arterial partial pressure of oxygen; n, number.

Fig. S1. Positive End-Expiratory Pressure by patient group.

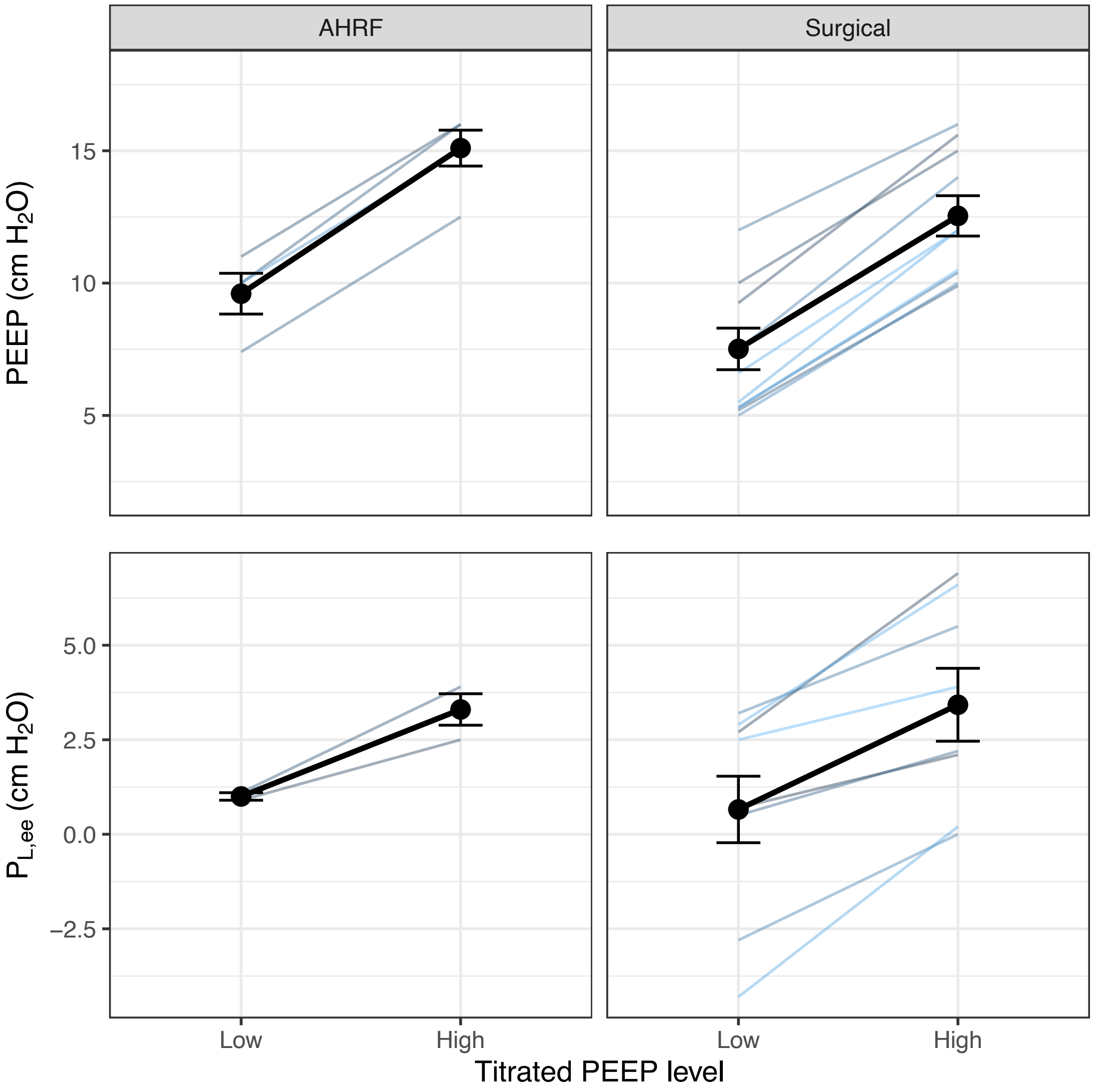

Individual patient trends (colored lines) alongside mean and standard error (black) for total (measured) positive end-expiratory airway pressure (top panel, n=16), and end-expiratory transpulmonary pressure (bottom panel, n =12) at baseline, by group.

AHRF, acute hypoxemic respiratory failure; PEEP, positive end-expiratory airway pressire; $P_{L,ee}$, end-expiratory transpulmonary pressure.

Fig. S2. Relationship between the level of diaphragm neurostimulation and magnitude of inspiratory effort.

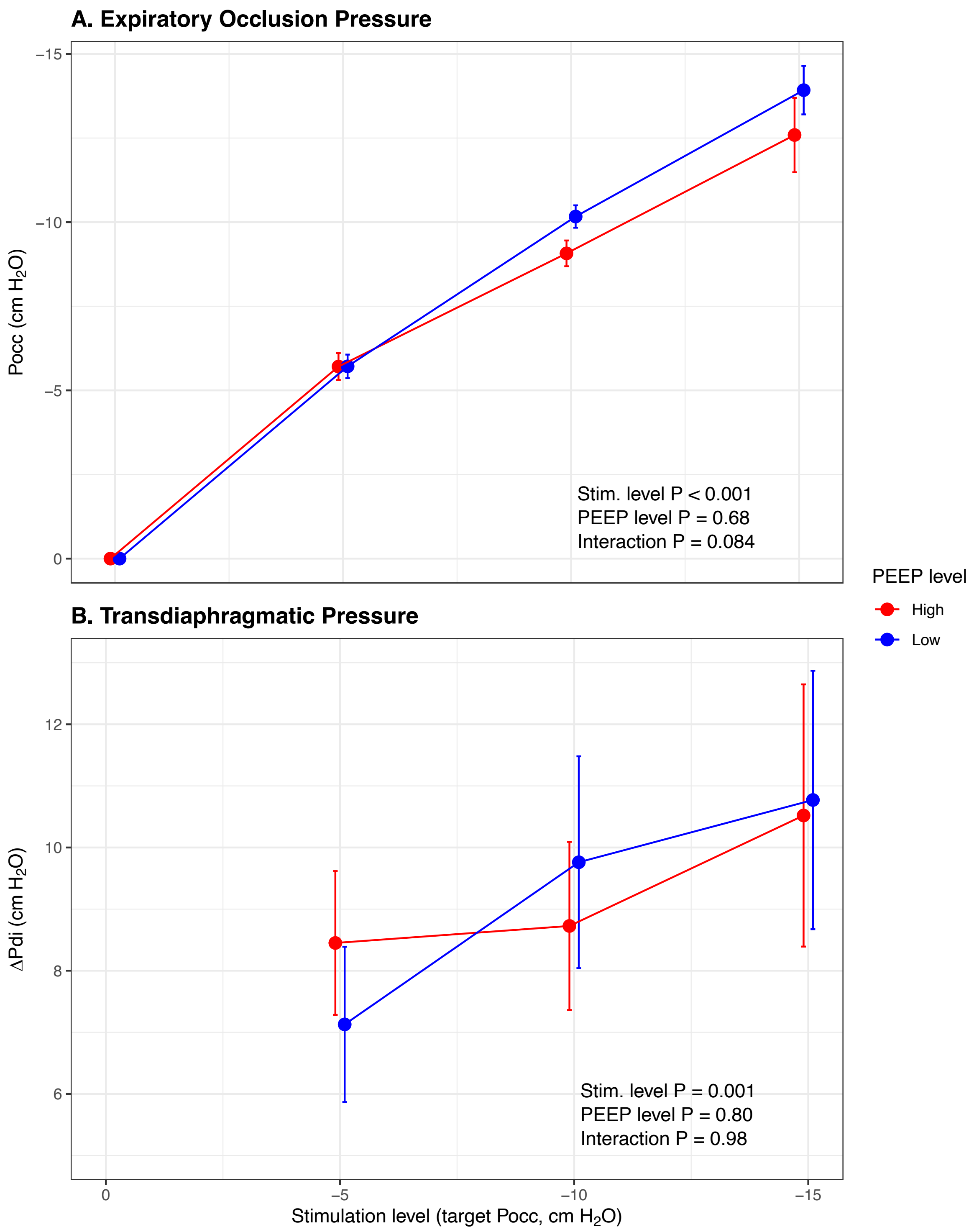

Mean and standard error for measured expiratory occlusion pressure (panel A, n=16) and non-occluded transdiaphragmatic pressure (panel B, n=9).

Pocc, expiratory occlusion pressure; P_{di}*, transdiaphragmatic pressure; Stim., stimulation; PEEP, positive end-expiratory pressure.*

Fig. S3. Effect of diaphragm neurostimulation on respiratory mechanics by patient group.

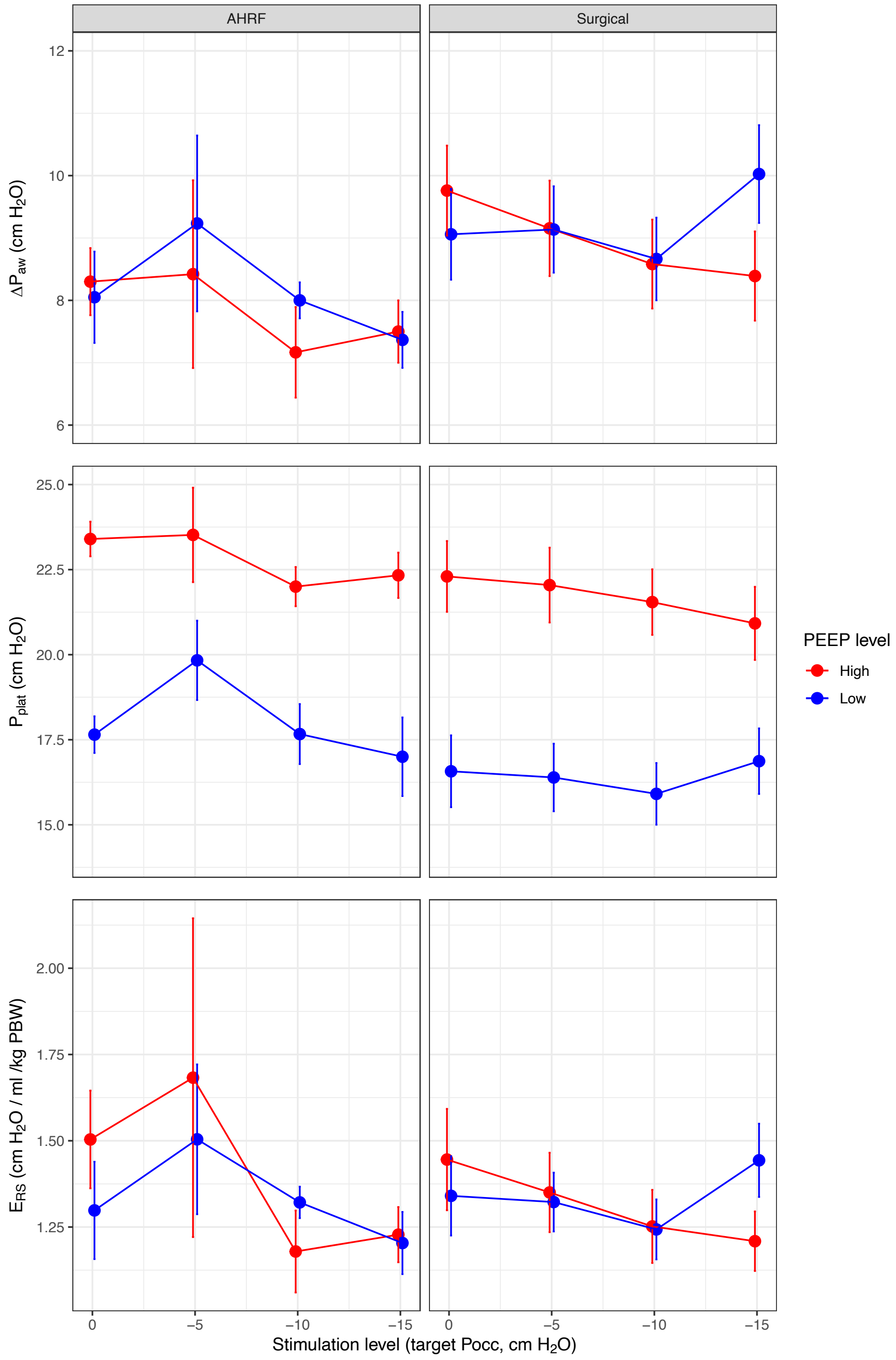

Mean and standard error for static driving pressure (top; n=16), plateau pressure (middle; n=16) and normalized respiratory system elastance (bottom; n=16) by group (AHRF right, surgical right).

P value for interactions between stimulation level and group for static driving pressure, p=0.96; plateau pressure, p=0.88; and normalized respiratory system elastance, p=0.84.

ΔP_{aw}, static driving pressure; P_{plat}, plateau pressure; E_{RS}, normalized respiratory system elastance; PBW, predicted body weight; PEEP, positive end-expiratory pressure; Pocc, end-expiratory occlusion pressure.

Fig. S4. Effect of diaphragm neurostimulation on respiratory mechanics at high and low PEEP for individual patients.

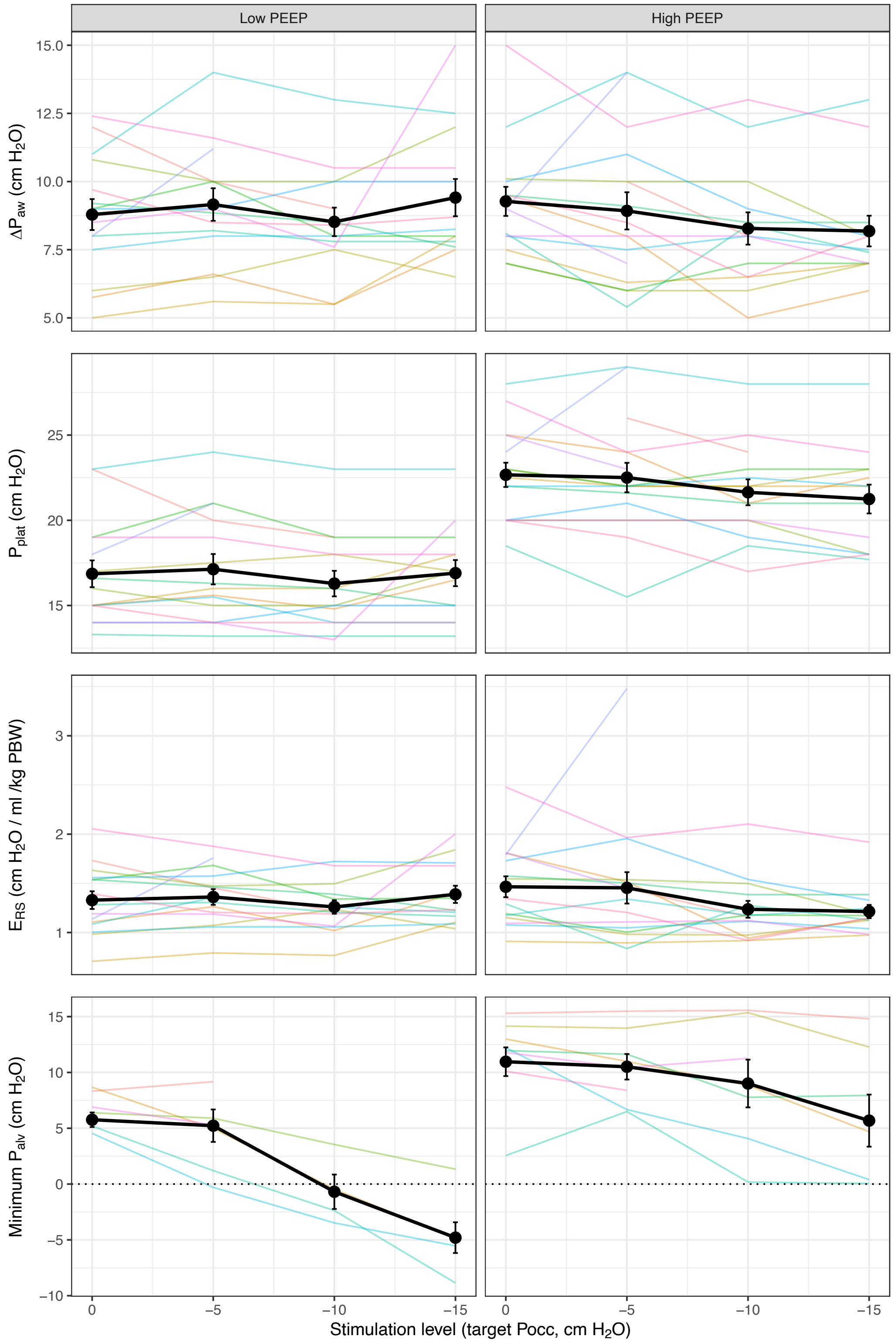

Individual patient trends (colored lines) alongside mean and standard error (black) for; static driving pressure, plateau pressure, normalized respiratory system elastance and estimated minimum alveolar pressure, at low and high PEEP.

ΔP_{aw}, static driving pressure; P_{plat}, plateau pressure; E_{RS}, normalized respiratory system elastance; PBW, predicted body weight; P_{alv}, alveolar pressure; PEEP, positive end-expiratory pressure; Pocc, end-expiratory occlusion pressure.

Fig. S5. Effect of diaphragm neurostimulation on measured dynamic transpulmonary driving pressure.

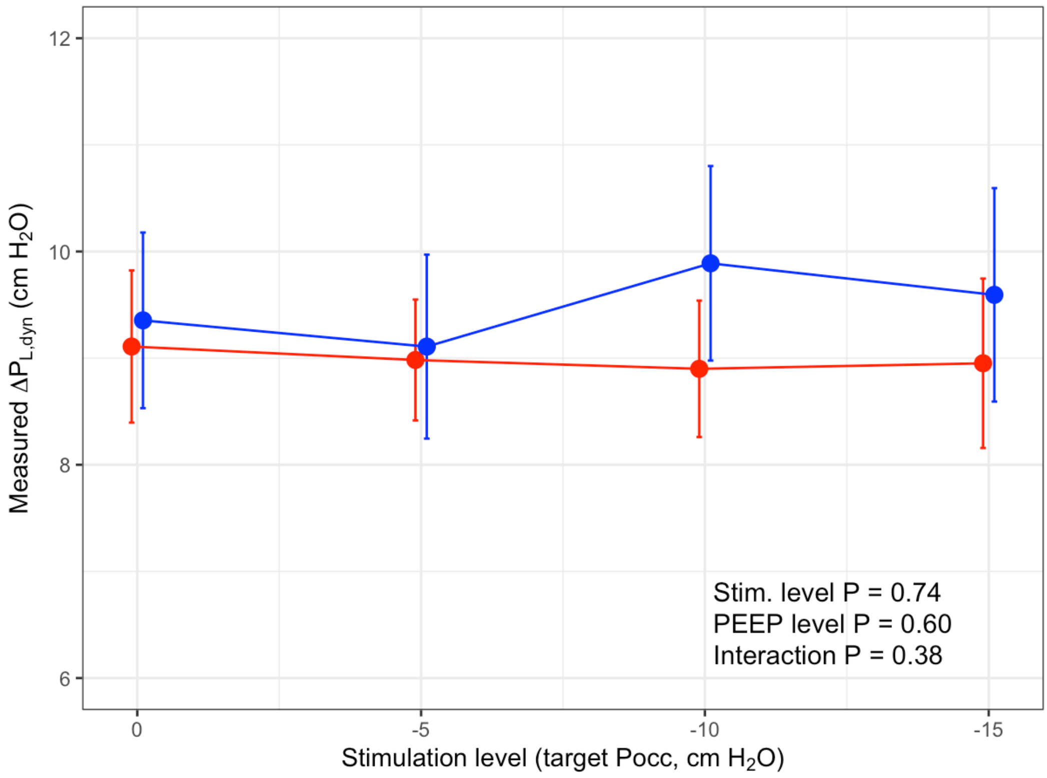

Mean and standard error for dynamic transpulmonary driving pressure (n=12) as measured by esophageal manometry.

$\Delta P_{L,dyn}$, dynamic transpulmonary driving pressure; PEEP, positive end-expiratory pressure; Pocc, end-expiratory occlusion pressure.

Fig. S6. Effect of diaphragm neurostimulation on gas exchange.

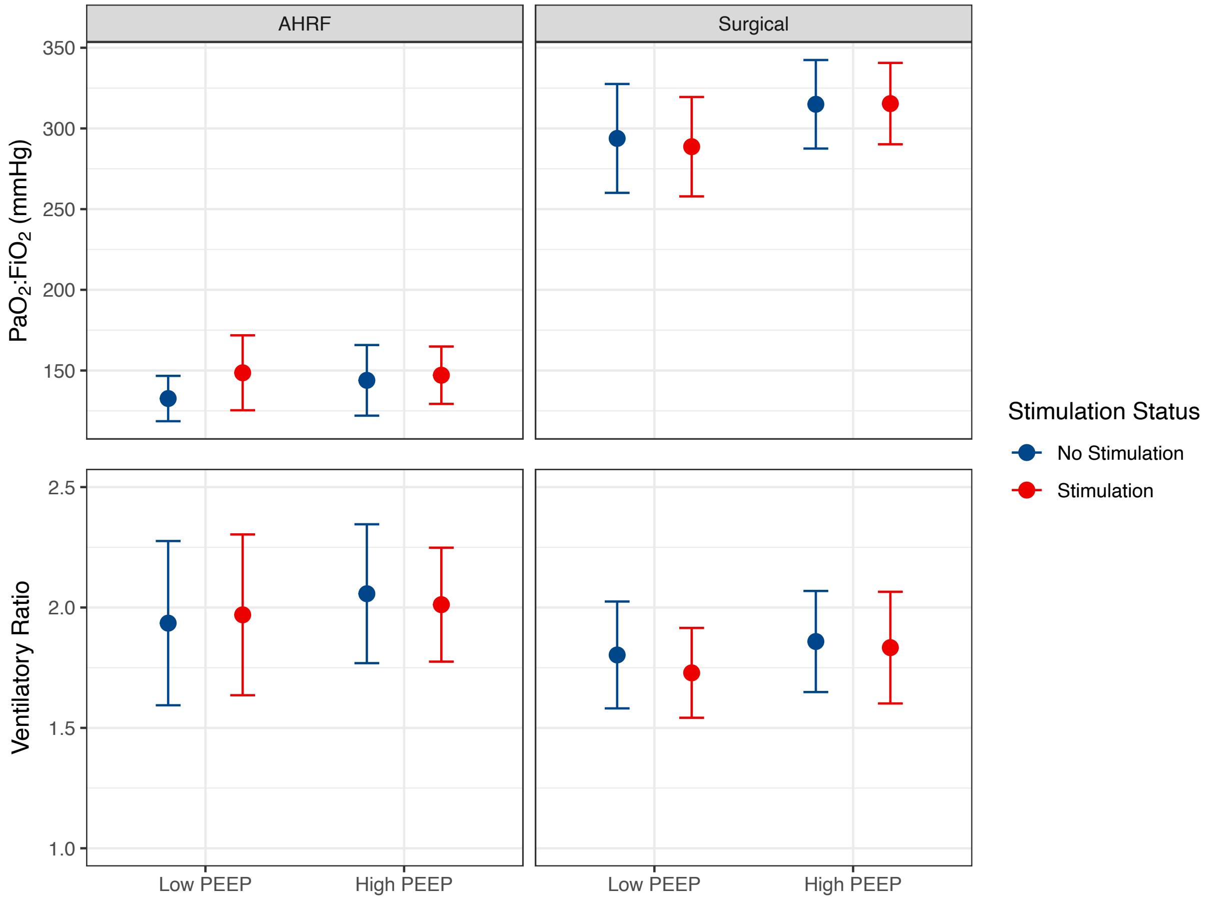

Mean and standard error for PaO_2/FiO_2 and ventilatory ratio by group (n=15).

P value for the effect of stimulation (0.17 and 0.39), PEEP (0.50 and 0.38) and PEEP-stimulation interaction (0.19 and 0.60) on PaO_2/FiO_2 and Ventilatory Ratio respectively.

Participants receiving extracorporeal membrane oxygenation are excluded from this analysis.

PaO_2, arterial partial pressure of oxygen; FiO_2, fraction of inspired oxygen; AHRF, acute hypoxemic respiratory failure; PEEP, positive end-expiratory pressure.

Fig. S7. Effect of diaphragm neurostimulation on distribution of inflation during mechanical ventilation by patient group.

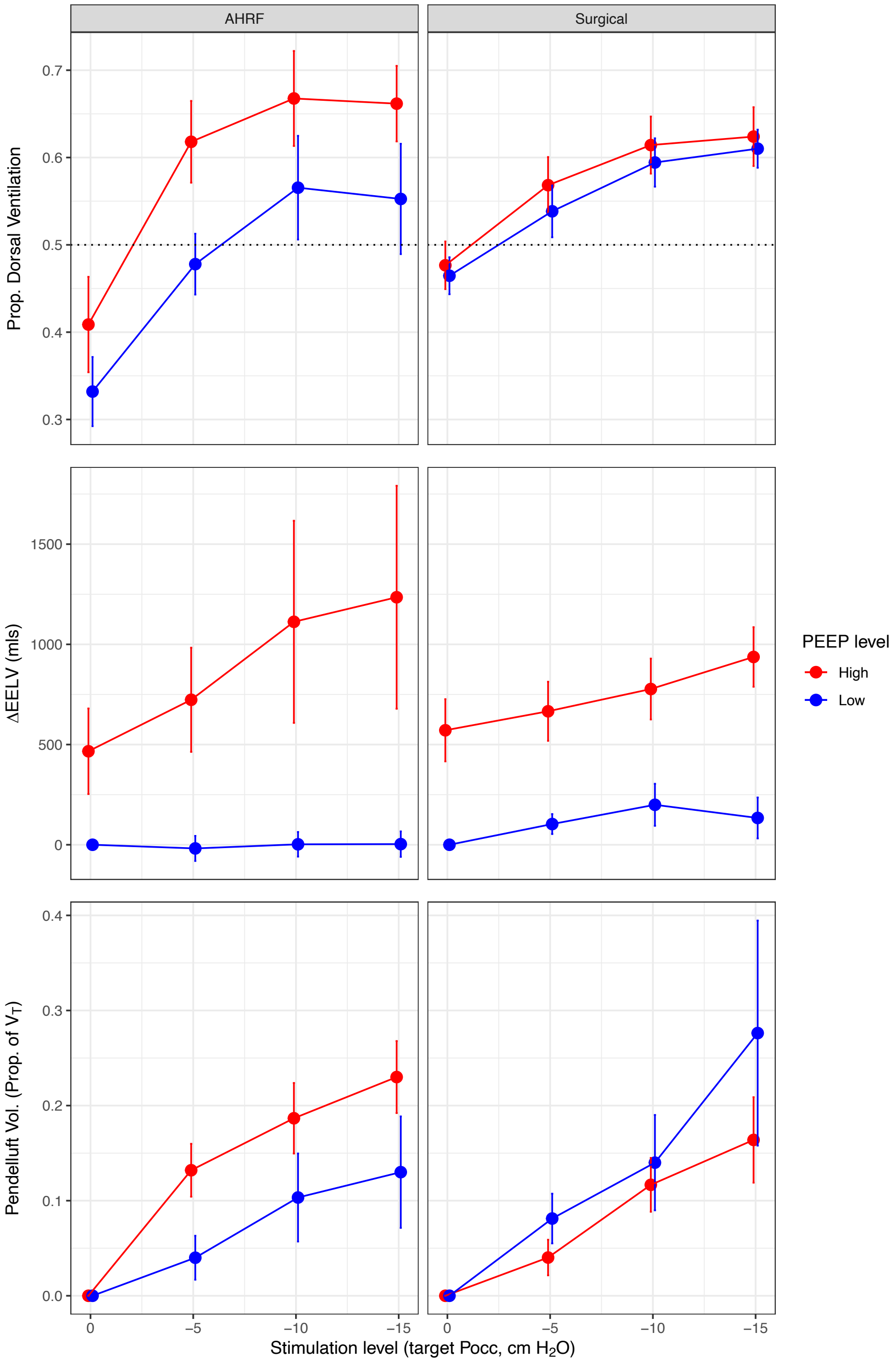

Mean and standard error bars for proportion of tidal ventilation distributed dorsally (top; n=15), change in end-expiratory lung volume (middle; n=15) and pendelluft volume as a proportion of tidal volume (bottom; n=15) by group (AHRF left, surgical right).

P value for the interaction between stimulation level and group for proportion of dorsal ventilation, p=0.012; change in end-expiratory lung volume, p=0.91; and pendelluft volume, p=0.52.

AHRF, acute hypoxemic respiratory failure; Prop, proportion; ΔEELV, change in end-expiratory lung volume (reference; low PEEP, no stimulation); Vol, volume; V_T, tidal volume; PEEP, positive end-expiratory pressure; Pocc, end-expiratory occlusion pressure.

Fig. S8. Effect of diaphragm neurostimulation on distribution of inflation at high and low PEEP for individual patients.

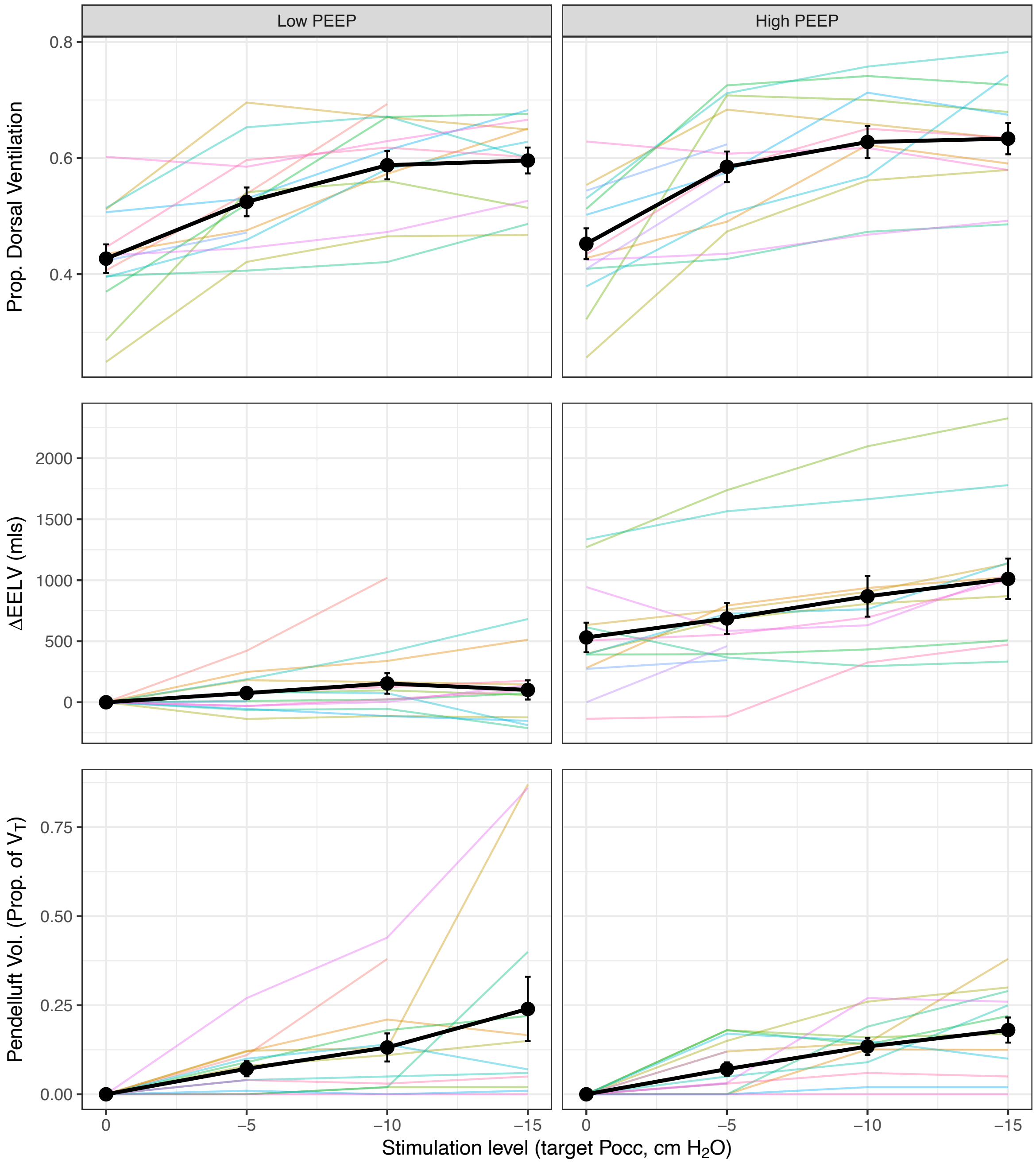

Individual patient trends (colored lines), alongside mean and standard error bars (black) for; proportion of ventilation distributed to the dorsal lung regions, change in end-expiratory lung volume, and pendelluft volume as a proportion of tidal volume, at low and high PEEP.

Prop., proportion; ΔEELV, change in end-expiratory lung volume (reference; low PEEP, no stimulation); Vol, volume; V_T, tidal volume; PEEP, positive end-expiratory pressure; Pocc, end-expiratory occlusion pressure.

Fig. S9. Comparison of intravascular versus transmural central venous pressure and mean pulmonary artery pressure.

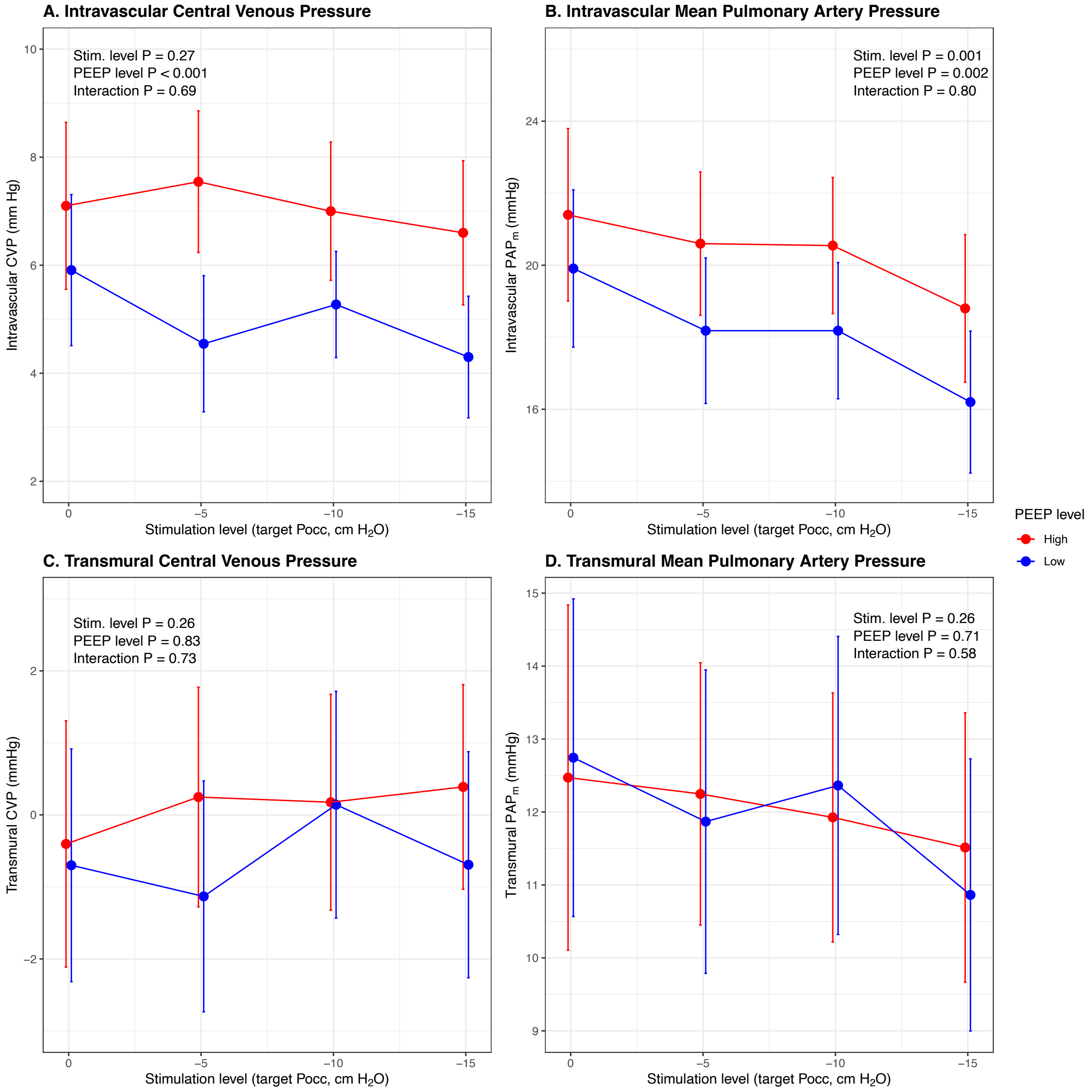

Mean and standard error bars for central venous pressure (left) and mean pulmonary artery pressure (right) referenced to atmospheric pressure (i.e. intravascular pressure; top, n=11) and mean esophageal pressure (i.e. transmural pressure; bottom, n=9).

CVP, central venous pressure; PAP_m, mean pulmonary artery pressure; Stim, stimulation; PEEP, positive end-expiratory pressure; Pocc, end-expiratory occlusion pressure.

Fig. S10. Effect of diaphragm neurostimulation on surrogate measures of right ventricular demand and performance.

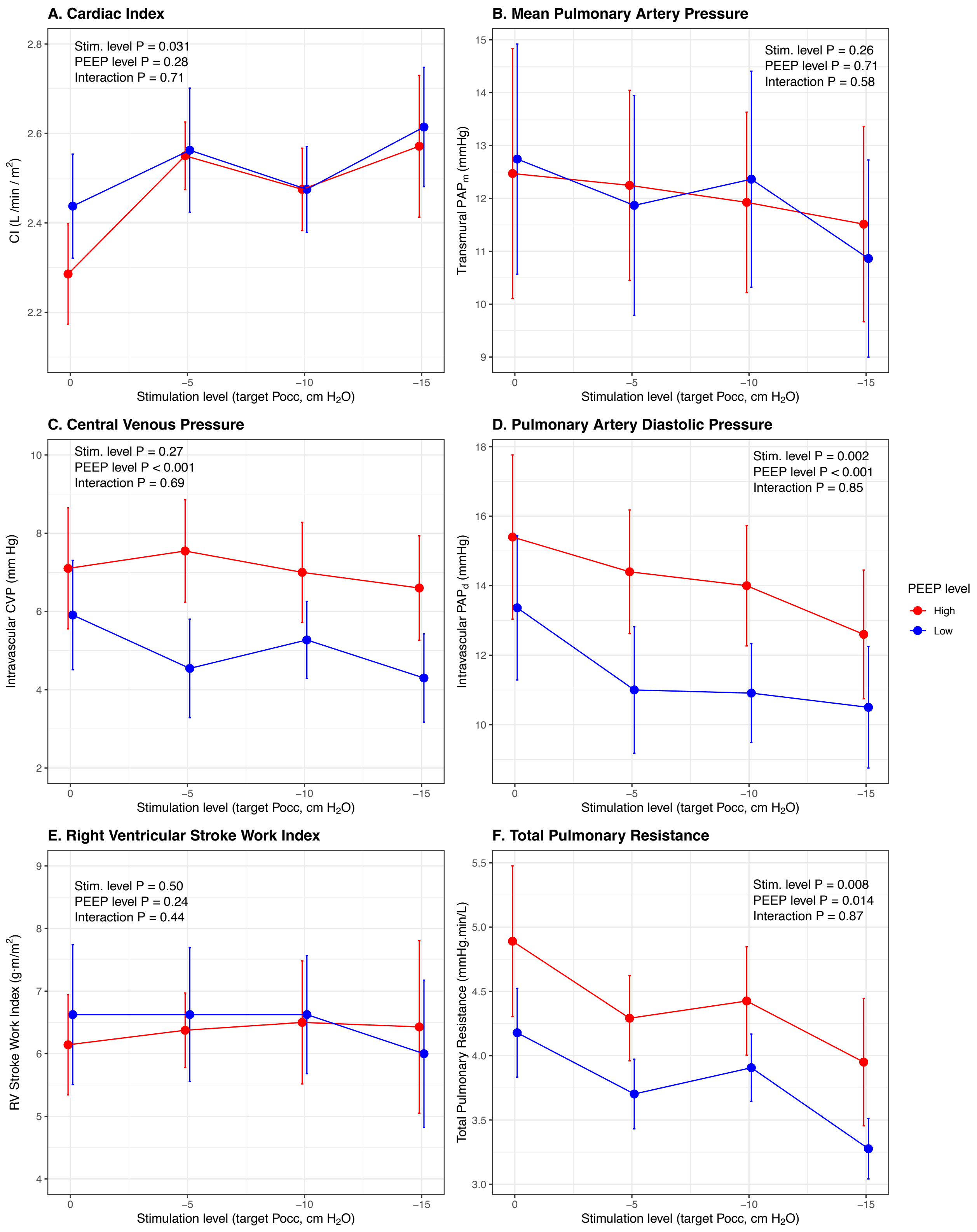

Mean and standard error bars for cardiac index (A; top left; n=8), mean pulmonary artery transmural pressure (B; top right; n=9), central venous intravascular pressure (C; mid left; n=11), pulmonary artery intravascular diastolic pressure (D; mid right; n=11), right ventricular stroke work index (E; bottom left; computed as [mean pulmonary artery pressure – central venous pressure] × stroke volume index × 0.0136; n=8), total pulmonary resistance (F; bottom right; computed as mean pulmonary artery intravascular pressure / cardiac output; n=8). All statistical models are adjusted for norepinephrine dose.

CI, cardiac index; PAP_m, mean pulmonary artery pressure; PAP_d, pulmonary artery diastolic pressure; RV, right ventricular; PEEP, positive end-expiratory pressure; Pocc, end-expiratory occlusion pressure.

Fig. S11. Effect of diaphragm neurostimulation on hemodynamics at high and low PEEP for individual patients.

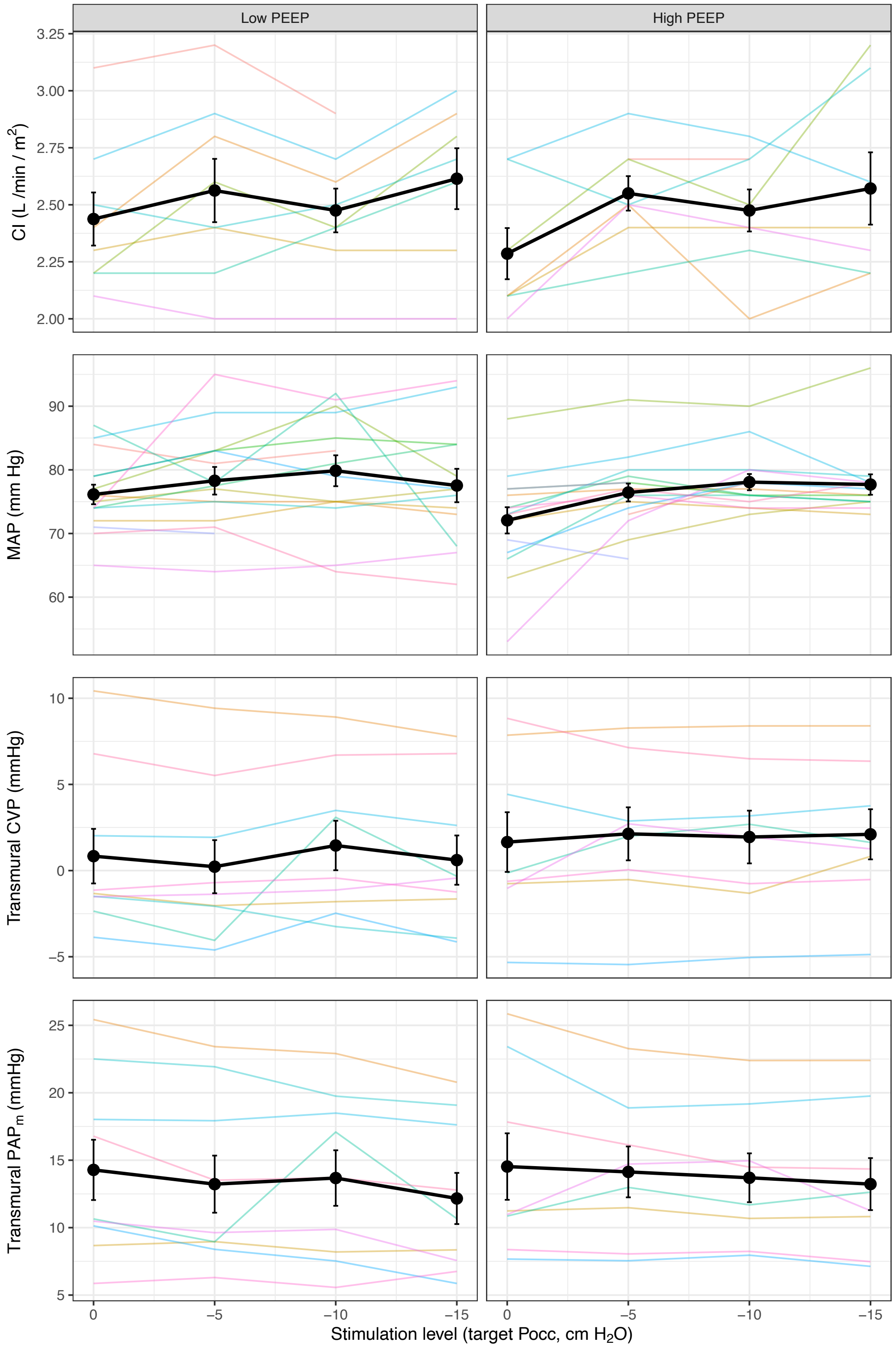

Individual patient trends (colored lines), alongside mean and standard error bars (black) for; cardiac index, mean arterial pressure, central venous transmural pressure and

mean pulmonary artery transmural pressure, at low and high PEEP. Central venous transmural pressure and mean pulmonary artery transmural pressure are computed by referencing intravascular pressures to mean esophageal pressure to account for differences in pleural pressure at different neurostimulation levels.

CI, cardiac index; MAP, mean arterial pressure; PAP_m*, mean pulmonary artery pressure; PEEP, positive end-expiratory pressure; Pocc, end-expiratory occlusion pressure.*

Fig. S12. Comparison of spontaneous breathing to passive ventilation without diaphragm neurostimulation, and passive ventilation with diaphragm neurostimulation in a single participant.

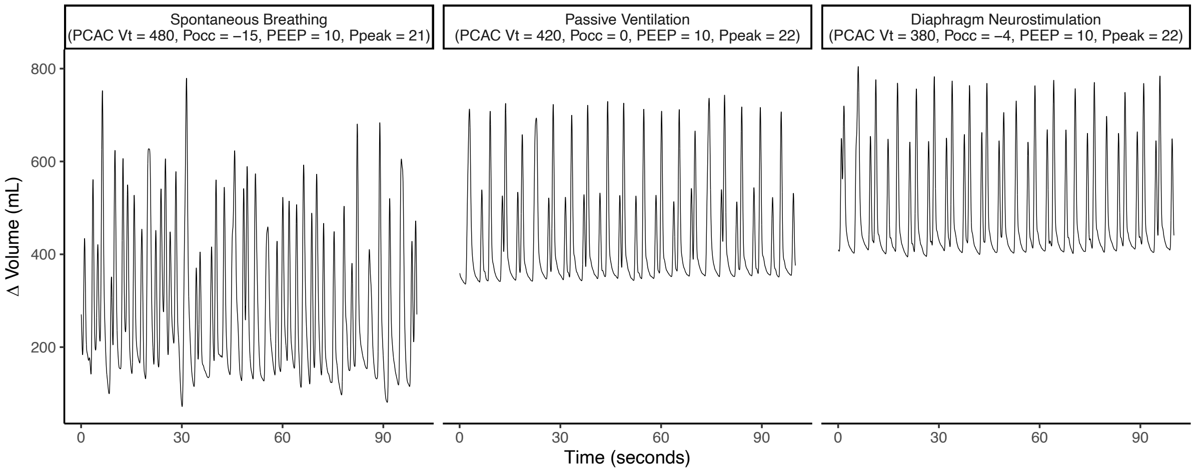

Change in volume during spontaneous breathing (left), passive ventilation (middle) and diaphragm neurostimulation (right) as assessed by EIT.

EIT, electrical impedance tomography; PCAC, pressure control assist control; Vt, tidal volume; Pocc, end-expiratory occlusion pressure; PEEP, positive end-expiratory pressure; Ppeak, peak airway pressure.
